# Two Capacitive Micro-Machined Ultrasonic Transducers for Wind Speed Measurement

**DOI:** 10.3390/s16060814

**Published:** 2016-06-02

**Authors:** Gia Thinh Bui, Yu-Tsung Jiang, Da-Chen Pang

**Affiliations:** Department of Mechanical Engineering, National Kaohsiung University of Applied Sciences, 415 Jiangong Road, Sanmin District, Kaohsiung 807, Taiwan; 1100403116@gm.kuas.edu.tw (G.T.B.); x59262002@gmail.com (Y.J.)

**Keywords:** capacitive micro-machined ultrasonic transducer (CMUT), wind speed measurement, amplitude, time-of-flight

## Abstract

This paper presents a new wind speed measurement method using a single capacitive micro-machined ultrasonic transducer (CMUT). The CMUT was arranged perpendicular to the direction of the wind flow, and a reflector was set up a short distance away, facing the CMUT. To reduce the size, weight, cost, and power consumption of conventional ultrasonic anemometers this study proposes two CMUT designs for the measurement of wind speed using either the amplitude of the signal or the time of flight (TOF). Each CMUT with a double array element design can transmit and receive signals in five different operation modes. Experiments showed that the two CMUT designs utilizing the TOF were better than those utilizing the amplitude of the signal for wind speed measurements ranging from 1 m/s to 10 m/s, providing a measurement error of less than 0.2 m/s. These results indicate that the sensitivity of the TOF is independent of the five operation modes.

## 1. Introduction

Capacitive micro-machined ultrasonic transducers (CMUTs) emerged as an interesting alternative to piezoelectric transducers in the mid-1990s [1,2]. Over the past two decades, several research studies have been conducted to explore the fabrication [3,4], characterization [5], and modeling [6] of CMUTs. Our goal in conducting this research was to develop new CMUT array designs that are useful for measuring wind speed through the use of a single CMUT.

The measurement of fluid speed, particularly wind speed, using ultrasonic transducers has been found to have important scientific and industrial applications [7,8]. Wind speed measurements can be applied not only in industrial fields, but also for the purpose of environmental monitoring and the control of various systems, such as indoor air conditioning systems, weather forecasting systems, and other sensors [9,10]. Some applications, such as those providing an evaluative measurement of wind speed for wind power generation, require measurements with low levels of uncertainty [11]. To design an instrument for measuring wind speed with measurement uncertainty that is as low as possible, a detailed study of the sources of measurement uncertainties and their propagation was conducted [12].

A common approach for determining wind speed is to measure the time-of-flight (TOF) by means of an ultrasonic signal, where the TOF is defined as the time required for an ultrasonic wave to travel from its source of transmission to the receiving transducer [13]. In the existing literature, several measurement techniques based on TOF estimates have been presented and discussed, including the threshold detection (TH), phase difference (PD), cross-correlation, maximum likelihood estimation (MLE), Kalman filter and wavelet transform techniques [14]. The TH and PD techniques are straightforward when compared with other techniques that make use of signal correlation [15]. However, an estimation of the TOF requires the consideration of factors such as attenuation of the medium, random noise, and reflections, which lower the signal noise ratio (SNR) and increase uncertainty to high levels.

To develop a minimally complex CMUT array for ultrasound transmissions and reception, some publications recommended an ultrasound flow measurement system with a transmitter and receiver on the same substrate as our system. Instead of using Doppler effects for flow measurement [16,17], we used the TOF method to determine wind velocity. In the comparison with the methods used in previous studies [18,19], our method also has the advantage of allowing for the quick calculation of results from simple equations.

In this paper, we propose two CMUT designs to measure wind speed, based on the TOF and amplitude of ultrasonic signals between transmission and reception. Specifically, we use only a single CMUT to yield a measurement of velocity with small spatial resolution of 10 mm between the reflector and transducer. The findings from this study can reduce the number of sensors needed, compared with previous systems [20,21,22]. Furthermore, the 3 mm × 3 mm CMUT presented in this study is also smaller than other ultrasonic anemometers that have been used previously [23]. Because of the smaller distance between the CMUT and reflector, the travel time is shorter and the CMUT’s sampling rate is higher.

The CMUTs for wind speed measurement have a novel design for the transmission and reception of signals in five different modes of operation. This approach also uses the available array element area efficiently. In this paper, the implementation of two new CMUT designs for wind speed testing is described, and the pulse-echo testing results are discussed. These results show that a single CMUT can be used for wind speed measurement with good accuracy. In addition, the whole system can be realized with a simple structure and low costs.

## 2. CMUT Design

**Figure 1:**
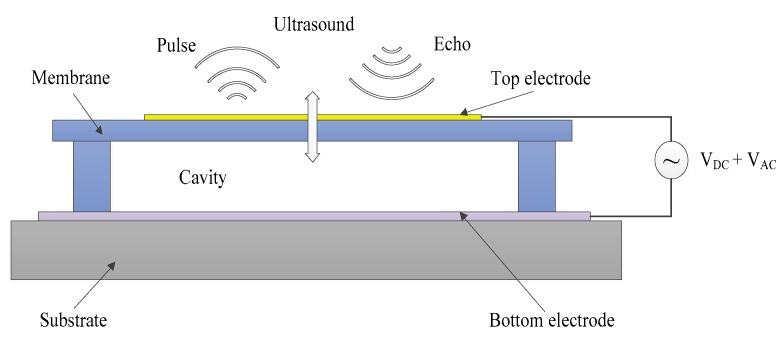
Cross-sectional schematic of a single CMUT cell.

The basic mechanical structure of a CMUT is similar to that of a capacitor with two parallel plates (see Figure 1). The membrane, filling layer, and side wall are all made of polymer, an epoxy-type photoresist (SU-8). The filling layer is designed to protect the gold top electrode from the ambience and external corrosion during regular operation. A thin indium tin oxide bottom electrode is adhered on a substrate and separated from the membrane by an air cavity. The geometric parameters of a CMUT are summarized in Table 1.

**Figure 2:**
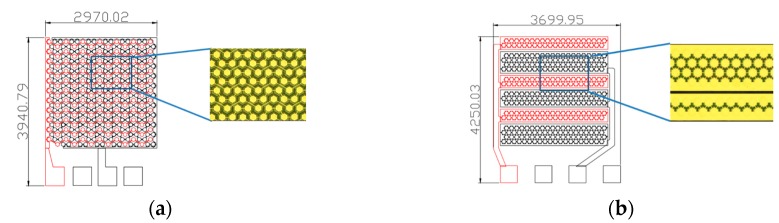
Diagram of two CMUT designs: (**a**) Wave-type; (**b**) Parallel-line type.

The aim of this research is to design new CMUTs with divergent characteristics. The transmission and reception modes of a single device can be operated separately so that the element can be adjusted individually to transmit and receive ultrasonic signals. The single device as designed can work with five operation modes using different ratio of transmission and reception cells. In this study, two CMUT designs are presented, as shown in Figure 2. Each one was arranged in a square area measuring 3 mm × 3 mm with a diameter and thickness of 140 and 5 µm, respectively, for each cell membrane and a cavity height of 2 µm. Each CMUT has two arrays of cells, called the majority and minority arrays. The majority array has more cells than the minority array, with a 2:1 ratio, in these designs. In the wave-type CMUT design, the array element is separated using 0.1 mm spacing to include 416 cells inside it, as shown in Figure 2a. Figure 2b shows the parallel-line type CMUT arranged in a rectangular array, with a majority array of 80 cells arranged in a 3 mm × 0.65 mm area and a minority array of 40 cells arranged in a 3 mm × 0.35 mm area.

## 3. Measurement Principle

**Figure 3:**
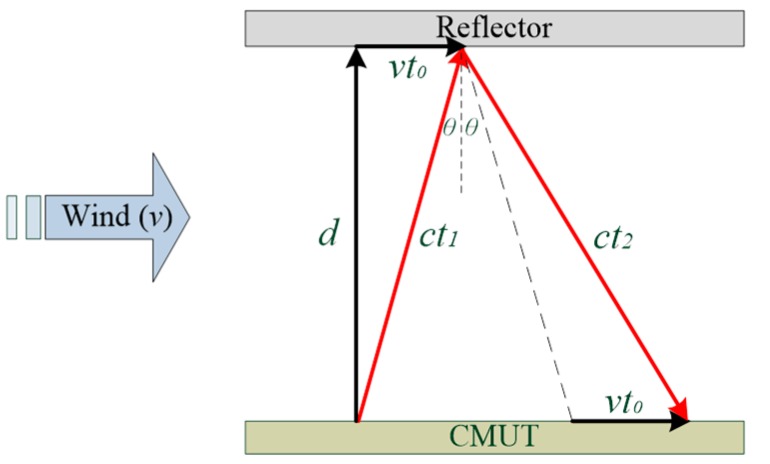
Wind speed measurement using the CMUT.

It is common to use two ultrasonic transducers for measuring wind speed by lining up the transmitting and receiving transducers at a specific angle. This study proposes a new measurement system that uses only one CMUT and one reflector within a small space as shown in Figure 3. In this design, the wind flows perpendicular to the ultrasound signal path between the CMUT and the reflector. The wind will change the ultrasound propagation by increasing the TOF and reducing the amplitude of the ultrasound signals. As shown in Figure 3, the red vector is the ultrasonic path when it is altered by the wind flow, *d* is the distance (*i.e.*, 10 mm) between the CMUT and the reflector, and θ is the angle between the transmitted path of the ultrasonic signal and the shifted condition of the wind direction.

### 3.1. Measuring Wind Velocity

In this study, a method of measuring wind velocity was developed based on the different measurements times of the transmission time of the ultrasonic signals. This measured time difference *Δt* is expressed as Equation (6), which is derived from Equations (4) and (5). The latter equations are themselves derived from Equations (2) and (3) in terms of the velocity of wind *v* being much lower than sound speed *c*. The time difference of the ultrasonic signals transmitted *Δt*_1_ and echoed *Δt*_2_ were determined by substituting the measured transmitted time *t*_1_ and echo time *t*_2_. The transmission time of the ultrasonic signal *t*_0_ to the reflector without wind is measured as presented in Equation (1):

(1) $$t_{0}=\frac{d}{c}$$

(2) $$t_{1}=\frac{\sqrt{d^{2}+{\left({\text{vt}}_{0}\right)}^{2}}}{c}=t_{0}\sqrt{1+{\left(\frac{v}{c}\right)}^{2}}=t_{0}+\frac{1}{2}{\left(\frac{v}{c}\right)}^{2}t_{0}+\frac{1}{2}{\left(\frac{v}{c}\right)}^{4}t_{0}+\ldots +\frac{1}{2}{\left(\frac{v}{c}\right)}^{n}t_{0}$$

(3) $$t_{2}=\frac{\sqrt{d^{2}+{\left(2{\text{vt}}_{0}\right)}^{2}}}{c}=t_{0}\sqrt{1+{\left(\frac{2v}{c}\right)}^{2}}=t_{0}+\frac{1}{2}{\left(\frac{2v}{c}\right)}^{2}t_{0}+\frac{1}{2}{\left(\frac{2v}{c}\right)}^{4}t_{0}+\ldots +\frac{1}{2}{\left(\frac{2v}{c}\right)}^{n}t_{0}$$

(4) $$\Delta t_{1}=t_{1}-t_{0}\approx \frac{1}{2}{\left(\frac{v}{c}\right)}^{2}t_{0}$$

(5) $$\Delta t_{2}=t_{2}-t_{0}\approx \frac{1}{2}{\left(\frac{2v}{c}\right)}^{2}t_{0}$$

(6) $$\Delta t=\Delta t_{1}+\Delta t_{2}=\frac{5}{2}{\left(\frac{v}{c}\right)}^{2}\frac{d}{c}$$

Temperature and humidity affect the density of the air, causing changes in the velocity of the ultrasound signal [24,25,26]. If the CMUT is used at room temperature with less than 1 °C variation, the ultrasound velocity change is within 0.2%. The humidity effect on ultrasound velocity is even smaller. The ultrasound velocity change is only 0.05% if the temperature is constant and humidity changes remain within 10%.

### 3.2. Wind Speed Measurement System Configuration

**Figure 4:**
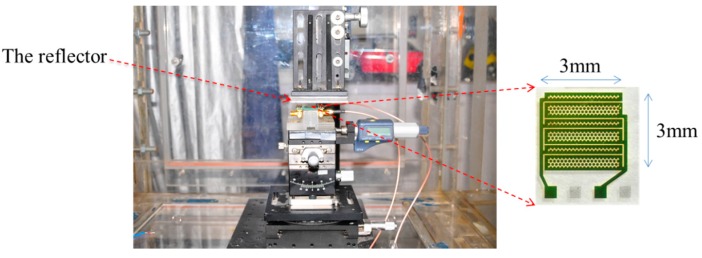
Photograph of the wind speed measurement configuration.

A wind speed measurement system was initiated for basic measurements, as shown in Figure 4. The measurement box had the dimensions of 120 cm × 60 cm × 60 cm. On cross-sections perpendicular to the wind flow direction, the wind velocity was determined on a grid. A transducer was installed on the calibration system. The wind speed tests were performed in the laboratory at a temperature of 25 °C ± 0.5 °C and humidity of 60% ± 3%. The effects of temperature and humidity on ultrasound velocity were very small. The environment uncertainty was approximately 0.2%.

**Figure 5:**
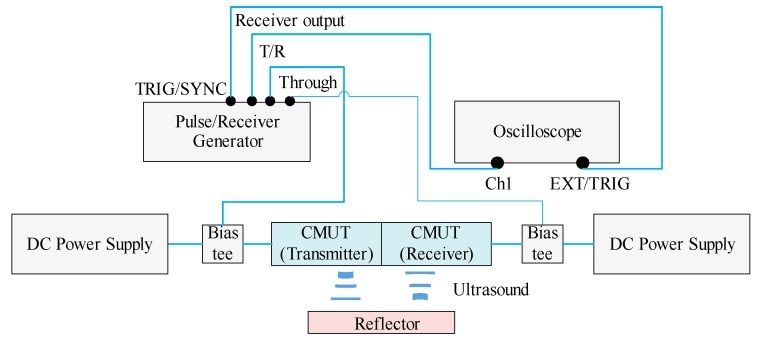
Setup for wind speed experiment.

Figure 5 shows the setup for the wind speed experiment. The CMUT was powered by DC 100 V and AC 300 V power sources, which produced the ultrasonic pulse signals used in the experiment. A generator (DPR 300 Pulse/Receiver, JSR Ultrasonics, Pittsford, NY, USA) transmits a short pulse, which has an amplitude of 0–475 V. A variable DC bias voltage of 0–300 V (N5751A DC Power Supply, Keysight Technologies, Santa Rosa, CA, USA) is superimposed on the transient voltage. The receiver signals were obtained with an oscilloscope (DPO 4104B Digital Phosphor Oscilloscope, Tektronix, Beaverton, OR, USA) connected to the CMUT. Any TOFs obtained in the measurements were recorded and transmitted to a computer.

**Figure 6:**
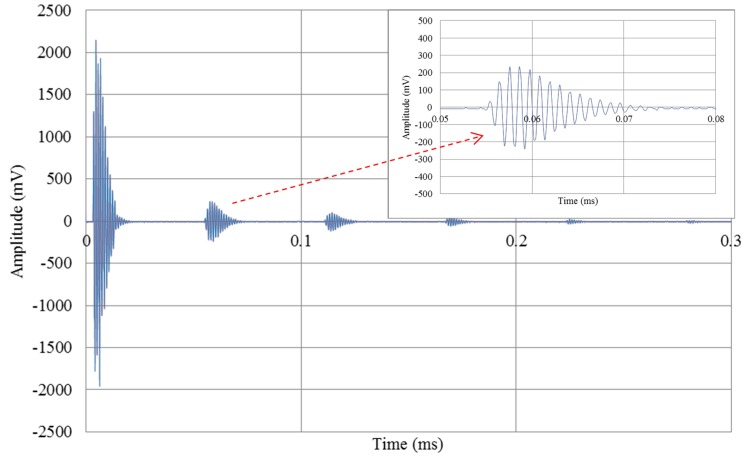
Time response of pulse-echo signal in full/full mode.

Figure 6 shows the received ultrasonic signal’s amplitude. The measurement system starts its operation with a pulse-wave transmitted and received by the transducer, which is propagated in the medium. The time needed for the detection of the received ultrasonic signal was determined using a reference threshold. From this detection, a data acquisition process utilizing the transmitted and received signals was conducted to determine any and all detection times. Then, an analysis of the signals led to estimates of the PD between the transmitted and received signals. The number of cells and the signal amplitudes of the two CMUT designs in five operation modes are shown in Table 2.

The operation of the transducer is based on the ratio of transmission and reception cells. The experimental measurements have five modes: full transmission/full reception, majority transmission/majority reception, majority transmission/minority reception, minority transmission/majority reception, and minority transmission/minority reception. The full transmission/full reception mode had the maximum received signals and the highest sensitivity. The minority transmission/majority reception mode, had fewer transmitted signals than the majority transmission/majority reception mode but still received some signals and was considered better and relatively more efficient.

## 4. Experimental Results

**Figure 7:**
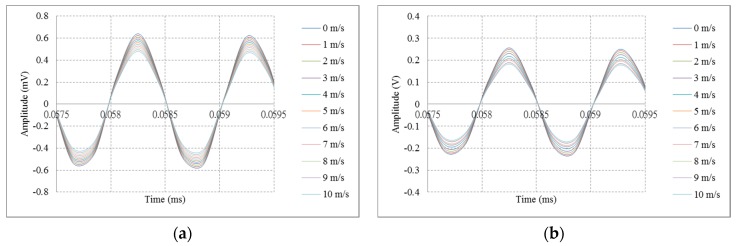
Increasing attenuation of the echo signal by wind speeds from 1 m/s to 10 m/s: (**a**) The full transmission/full reception mode results; (**b**) The minority transmission/majority reception mode results.

In this experiment the transducer has a 90° directivity angle and a reflecting surface set at a distance of 10 mm. The maximum signal of the first echo was used as an observation point, whereby it was found that the attenuation of the signal increases as the wind speed increases. The peak time increases slowly along with the increasing wind speed, but the operation time of the first echo and of the resonance frequency does not change. This study took advantage of the time difference between the increasing attenuation of the echo signals to determine wind speed changes. As shown in Figure 7, the experiments measured wind speeds from 1 m/s to 10 m/s and the results of the full transmission/full reception mode and minority transmission/majority reception showed that the values of the maximum points of the echo signal change when the wind speed increases.

### 4.1. Using Amplitude of Signal to Measure Wind Speed

**Figure 8:**
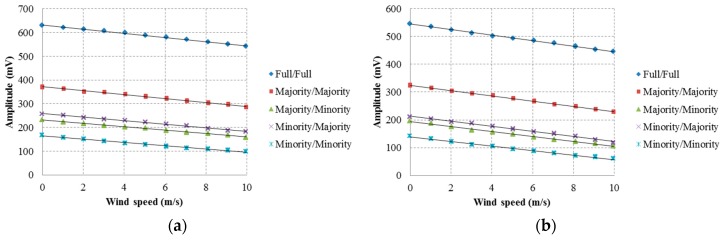
Effect of wind speed on measurement results: (**a**) Wave-type CMUT design; (**b**) Parallel-line type CMUT design.

As shown in Figure 8a, the wave-type transducer design was tested. The experimental results of the five modes were affected by the wind speed, with sensitivity of magnitude signals and a standard deviation of error as shown in Table 3. Figure 8b shows the results achieved when using the parallel-line type ultrasonic transducer design for measurement. The measurement results of the five modes were affected by the wind speed, with attenuation sensitivity of magnitude signals and a standard deviation of error as shown in Table 3. The results were based on the least square method to draw the best fit line. From the results, it can be concluded that the strength of the wind speed had a direct effect on the amplitude of the ultrasonic signals. The ultrasonic echo signals of the two types of transducers were smaller when the attenuation of the wind speed increased. The measurements of the two transducers in full transmission/full reception mode demonstrated high sensitivity significance. It was found that the minority transmission/majority reception mode had a larger signal than in majority transmission/minority reception mode.

### 4.2. Using Differences in TOF’s to Measure Wind Speed

**Figure 9:**
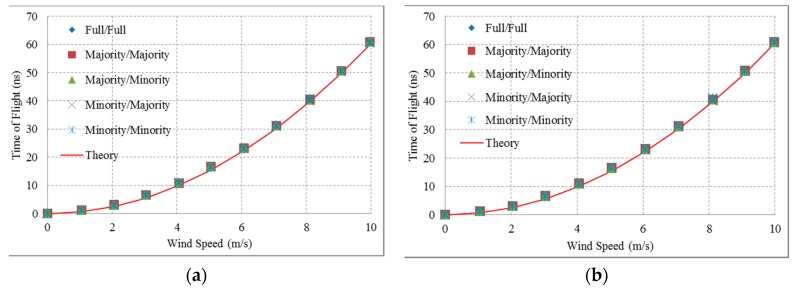
Comparison of the results from the theoretical calculation and the experimental measurements according to different TOFs: (**a**) Wave-type CMUT design; (**b**) Parallel-line type CMUT design.

In this study, the transducers used self-transmitted and self-received signals sent over a reflector distance of 10 mm to measure wind speed. The influence of the wind causes the point of a signal’s arrival on the reflector to be offset by a distance of *vt*, and the wind effect on the speed of an ultrasound signal is offset again after reflection as shown in Figure 3. The transducers were used in experiments to measure wind speeds ranging from 1 m/s to 10 m/s. With greater wind speeds, the time required to receive signals increases. The TOF can be calculated from the experimental results and compared with the theoretical results as shown in Figure 9.

There are significant errors evident when the wind speed is small. This contradicts the presented theoretical values. The results utilized the least square method to draw the second order curve fitting. Table 3 shows the wind speed measurement error using a difference in TOF that is slightly lower than the amplitude of the signal.

## 5. Conclusions

In this paper, two designs of a CMUT with five modes that can be used to measure the velocity of wind blowing perpendicular to the CMUT were presented. In addition, analyses of the ultrasound signal amplitudes and the TOF differences due to different wind speeds were conducted. The problems with the measurement range for the TOF differences and signal amplitudes of the continuous-wave CMUT were compared. The two CMUT designs that used the TOF method did not show much of a difference in terms of sensitivity and error. The TOF determination method can be used to measure wind speeds without calibration. Also, it was observed that the full transmission/full reception mode was the best choice in terms of providing greater sensitivity and less uncertainty in wind speed measurement when using the amplitude of signal method. The minority transmission/majority reception mode is proposed for good energy efficiency since the design can be selected for signal transmission. The parallel-line type design produced results that were only slightly better than those of the wave-type design. Thus, the two CMUT designs can be used to build a wind speed measurement system that is independent of the five modes and has the advantages of high accuracy, low cost, and easy portability, as well as great potential for future applications.

## Figures and Tables

**Table 1:** Mechanical dimensions of a CMUT cell.

Parameter	Measurement (μm)
Membrane diameter	140
Membrane thickness	5
Sidewall width	10
Cavity height	2
Bottom electrode thickness	0.05
Top electrode diameter	108
Top electrode thickness	0.15

**Table 2:** Number of cells and signal amplitudes of the two CMUT designs in the five operation modes.

Mode	Wave-Type Design		Parallel-Line Type Design	
	Transmission/Reception (Cell Number)	Signal (mV)	Transmission/Reception (Cell Number)	Signal (mV)
Full transmission/Full reception	416/416	634	360/360	546
Majority transmission/Majority reception	274/274	362	240/240	324
Majority transmission/Minority reception	274/142	226	240/120	196
Minority transmission/Majority reception	142/274	244	120/240	214
Minority transmission/Minority reception	142/142	158	120/120	140

**Table 3:** The sensitivities and standard deviation errors of measurement for the two CMUT designs.

Designs	Modes	Amplitude		TOF	
		Sensitivity (mV/ms^−1^)	Error (m/s)	Sensitivity (ns/(ms^−1^)^2^)	Error (m/s)
Wave-type CMUT	Full/Full	8.807	0.198	0.617	0.153
	Majority/Majority	8.379	0.254	0.616	0.154
	Majority/Minority	7.161	0.235	0.614	0.168
	Minority/Majority	7.555	0.272	0.617	0.161
	Minority/Minority	6.846	0.330	0.616	0.165
Parallel-line type CMUT	Full/Full	9.906	0.189	0.618	0.155
	Majority/Majority	9.552	0.180	0.616	0.156
	Majority/Minority	8.944	0.249	0.615	0.170
	Minority/Majority	9.166	0.241	0.617	0.165
	Minority/Minority	8.171	0.288	0.617	0.172
